# Sex-specific hormone signatures in four fish species with divergent reproductive strategies

**DOI:** 10.3389/fendo.2026.1923972

**Published:** 2026-10-02

**Authors:** Freya Adele Pappert, Olivia Roth

**Affiliations:** Marine Evolutionary Biology, Zoological Institute, Christian-Albrechts-Universität Kiel, Kiel, Germany

**Keywords:** endocrinology, male pregnancy, sex-roles, steroid hormones, syngnathid

## Abstract

**Introduction:**

Steroid hormones regulate diverse biological processes, including development, immunity and reproduction. Because biological sex, pregnancy, parental care, and mate competition are closely associated in most established model organisms, it remains unclear whether endocrine differences between males and females are primarily linked to gametic sex or to the reproductive roles performed by each sex. Syngnathid fish, the pipefishes, seadragons, and seahorses, provide a powerful system for disentangling these effects, because males, rather than females, carry the developing embryos. Species further differ in sexual selection and in the extent of paternal investment, ranging from externally attached eggs to incubation with an enclosed brood pouch.

**Methods:**

This study provides baseline hormonal profiles of key steroid and peptide hormones in three syngnathid species across the unique evolutionary parental care gradient of male pregnancy (*Nerophis ophidion, Syngnathus typhle, Hippocampus abdominalis*). We additionally included the female-pregnant livebearing fish Heterandria formosa as a comparative reference for a conventional separation of reproductive roles.

**Results:**

Sex-specific endocrine profiles broadly followed sexual selection differentials: species with stronger sexual heteromorphism, i.e., more extreme reproductive roles (*N. ophidion*), exhibiting stronger endocrine sexual heteromorphism. In contrast, species with higher parental care investment (*H. abdominalis, H. formosa*) show reduced endocrine differentiation between the sexes. The intermediate species S. typhle, which combines sex-role reversal with a moderately complex brood pouch, displayed tissue-specific patterns, with hormonal dimorphism retained primarily in the gonads.

**Discussion:**

By incorporating a cross-species comparative framework with both sex-role reversed and a conventional viviparous species, we can begin to understand which hormonal functions are conserved versus derived, and whether endocrine profiles are primarily aligned with biological sex or reproductive role, such as mate competition and parental care, thereby revealing how endocrine constraints and plasticity contribute to reproductive evolution.

## Introduction

Hormones play central roles in coordinating processes within and between living organisms, such as development, metabolism, and nutrient acquisition, sexual behaviour, and reproduction. They even mediate responses to environmental changes or communication between individuals (1–6). As such, variation in the expression and function of these components plays a major role in driving the evolution of morphology and life history (3, 7). While our understanding of steroid hormone function has expanded, it has primarily focused on species with conventional sex roles, in which females invest significantly in egg production and often take on the responsibility for rearing offspring (8). For instance, research suggests that sex hormones such as androgens can suppress the male immune system (9), whereas oestrogen has been linked to increased lifespan over the activation of telomerase activity and autophagy (10, 11). However, it is important to recognise that the designation of sex and sex roles in the animal kingdom is not fixed but rather fluid and dynamic. This variability raises the question of whether established relationships between hormones and sex-specific traits hold across systems with divergent reproductive strategies. In particular, it remains unclear how hormonal systems both respond to and potentially drive variation in mate choice, secondary sexual traits, mating behaviour, and parental care. Do shifts in sex roles or parental care strategies arise from underlying changes in endocrinological architecture, and how do hormones mechanistically contribute to the regulation of reproductive strategies? There is a need to adopt a comparative approach across species with diverse reproductive strategies, to not only enhance our understanding of steroid hormone function but also address the challenge of covariance between endocrine traits and life histories or fitness (4). Examining similarities and differences in endocrine profiles across species helps identify common principles underlying reproductive biology. This approach allows us to explore the diversity of adaptive solutions across taxa, thereby strengthening the generalizability of findings in comparative endocrinology and evolutionary biology (4, 7).

Addressing this requires a system in which biological sex, reproductive role, mating system, and parental care are naturally decoupled - a rare opportunity in most vertebrates. In systems with conventional reproductive roles, these traits are tightly linked, largely because hormonal pleiotropy allows single endocrine regulators to simultaneously coordinate physiology, morphology and behaviour. Therefore, it remains difficult to disentangle which factor drives hormonal variation when changes in one trait are inevitably altered in the others. Teleost fishes represent a highly diverse group of vertebrates with an exceptional range of reproductive strategies. In many species, males act as the primary caregivers, and variation in mating systems, reproductive roles, and parental care is widespread across the clade (12). In particular, the *Syngnathidae* family, which includes seahorses, pipefish, and seadragons, is home to some of the most dedicated male caregivers. They have evolved a range of brooding forms analogous to those found in mammalian maternal systems, with the female transferring eggs to the male before fertilization (13, 14). Fish endocrinology has long been a central topic in research, both for advancing fundamental knowledge and for supporting agricultural practices (15, 16). Despite the potential value of this model, endocrine control of fish reproduction remains poorly understood compared to mammals, as research has focused on only on commercial species or a few model species such as zebrafish or cichlid species (15). For example, 42% of published hormone studies on PRL have been conducted on cichlid species, whereas groups like the syngnathids - despite their unique reproductive features, including male pregnancy and sex-role reversal - remain comparatively understudied (17, 18).

The extent of male pregnancy varies depending on the pouch morphology between different species, ranging from simple egg attachment to fully enclosed pouches with placenta-like structures (13). Besides safeguarding the embryos, the brooding structure serves multifunctional roles during pregnancy, including osmoregulation, gas exchange, aeration, and in some more advanced species, nutrient provisioning (19, 20). This diversity in pregnancy complexity allows comparative investigation into how hormonal systems are associated with different levels of parental investment. In addition, this vibrant fish family exhibits an exceptional diversity of sex roles, ranging from conventional patterns (male-male competition and female choice) to reversed systems (female-female competition and male choice), and even flexible or plastic roles (21). Their mating systems are equally varied, including monogamy - where a male mates exclusively with a single female, as seen in many seahorses - polygynandry, with both sexes mating multiply (common in the genus *Syngnathus*), and polyandry, where females mate with multiple males (as in *Nerophis*) (22, 23). This variation allows comparisons of how hormonal profiles relate to behavioural roles and mating strategies independent of biological sex. Together, this diversity offers an exceptional comparative platform to disentangle the complex interplay between biological sex, reproductive strategy, parental care, and endocrine regulation. Rather than being merely biologically unusual, syngnathids represent a powerful natural experiment for understanding how reproductive strategies and hormonal systems co-evolve.

Male pregnancy in syngnathids does not involve a reversal of sex hormones, androgens continue to regulate spermatogenesis, and estrogens are involved in vitellogenesis and ovulation in females (24–26). However, progress in this family has been constrained by methodological limitations, including the difficulty of obtaining sufficient blood/plasma from the delicate fish for hormonal analyses (25, 27) and the sensitivity of detection methods was insufficient to reliably quantify hormones in such small sample volumes (17, 26). Many earlier investigations relied on techniques such as hypophysectomy or gonadectomy to examine steroid hormone function (18, 26). Although these approaches demonstrated that specific hormones are involved in reproduction, removal of the pituitary gland or gonads can induce systemic physiological changes that extend beyond the hormones of interest, making the conclusions drawn somewhat ambiguous. As a result, existing studies are often outdated, focusing on single hormones or entirely relying on inferred endocrine functions based on other teleost species, limiting comparability. We are therefore left to question how sex hormones and male pregnancy may have coevolved alongside reversed sex roles and different life-history strategies.

To that end, in our study, we aimed to assess how parental investment (e.g., brood pouch complexity) and sex-role dynamics (e.g., secondary sexual characteristics, courting behaviour) are linked to steroid and peptide hormone levels across a set of teleost species with varying degrees of parental investment and distinct reproductive strategies. We included the big-bellied seahorse *Hippocampus abdominalis*, with a fully enclosed vascularised brood pouch and conventional sex roles, the broadnosed pipefish *Syngnathus typhle* with a semi-sealed brood pouch and polygamous, and the straightnose pipefish *Nerophis ophidion* with simple egg attachment to the ventral side and polyandrous mating behaviour (28, 29), and finally the least killifish *Heterandria formosa* representing a teleost species with highly matrotrophic female pregnancy with a follicular placenta (**Figure 1**) (30, 31). We hypothesised that if hormonal profiles are primarily linked to biological sex, males should exhibit similar hormone profiles across species irrespective of brood pouch complexity and degree of sex-role reversal, and the same should apply to females. Alternatively, if hormones are associated with sex roles and parental investment, hormone concentrations should covary with either brood pouch complexity and/or mating system rather than with biological sex alone. Although our study species do not allow us to disentangle the independent effects of pouch evolution from mating system dynamics, any observed deviation from the sex-only baseline demonstrates that selection on reproductive strategy, whether via parental morphology or sex roles, modulates classic endocrine profiles.

**Figure 1:**
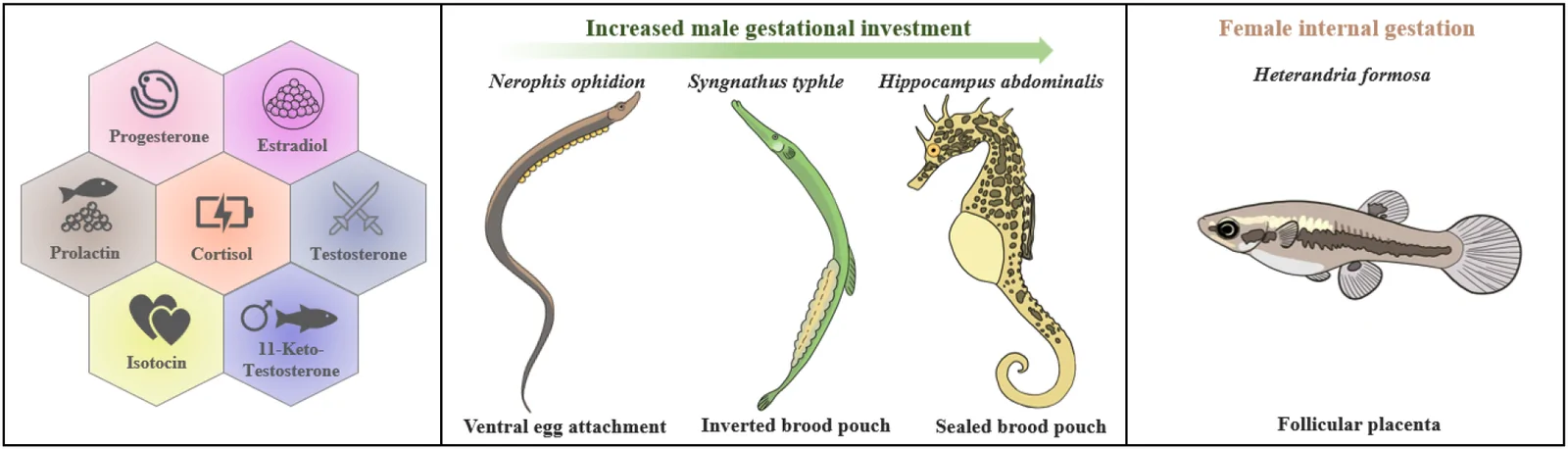
Graphical illustration (not to scale) showing, on the left side of the panel, the seven different hormones used in this study. These are followed by the three syngnathid species exhibiting male pregnancy, arranged according to increasing degree of paternal investment: *N. ophidion*, with simple egg attachment to the ventral trunk; *S. typhle*, with a semi-sealed brood pouch on the tail; and *H. abdominalis*, with a fully enclosed brood pouch. On the far right of the panel is *H. formosa*, a poeciliid species exhibiting high matrotrophic gestation via a follicular placenta (formed by the apposition of the maternal follicular epithelium to the embryonic surface).

We quantified hormone levels across multiple tissues, focusing on a panel of steroid and peptide hormones covering major endocrine pathways, including 17β-estradiol (E2), testosterone (T), 11-ketotestosterone (11KT or KT), progesterone (P), prolactin (PRL), isotocin (IT), and cortisol (CORT). Together, these hormones provide insights into the endocrine milieu underlying central aspects of reproduction and parental care. By integrating multiple endocrine pathways, our approach moves beyond the traditional focus on single hormones and allows us to assess how hormonal networks are associated with biological sex and divergent reproductive strategies.

## Materials and methods

### Animal husbandry and tissue sampling

Our study included four species, with both male and female adult individuals: *Hippocampus abdominalis* (n = 19; 10 males, 9 females), *Syngnathus typhle* (n = 24; 12 males, 12 females), *Nerophis ophidion* (n = 24; 12 males, 12 females), and *Heterandria formosa* (n = 14, 7 females, 7 males). Pipefish were collected from shallow coastal seagrass beds in the second half of April 2024, before the onset of the breeding season. The sampling site was located in the western Baltic Sea, in the bay of Orth, on the island of Fehmarn (54*°*26*′*52.03*′′* N, 11*°*02*′*54.84*′′* E). Snorkeling gear and hand fishing nets were used for collection. Immediately after capture, the fish were placed in an aerated transport container filled with ambient seawater and taken to the animal facility at Kiel University (CAU). Upon arrival, the fish were sorted by sex and transferred to separate 20L buckets containing a mixture of ambient seawater and water from the facility’s tanks, which helped the fish adjust from their natural environment to the facility conditions. The killifish were purchased in April 2024 from Zierfischtreff Teilenhofen in Germany, while the seahorses were purchased from Basel Zoo in Switzerland (February 2024).

All animals were housed in sex-separated aquaria at Kiel University under conditions emulating their natural habitats. *Syngnathus typhle* and *Nerophis ophidion* were maintained in 100 L tanks with a salinity of 16–18 PSU and water temperatures of 17–18 °C. *Hippocampus abdominalis* were kept at higher salinity (30–35 PSU) and temperatures of 16–18 °C. *Heterandria formosa* were housed in 20L freshwater tanks maintained at approximately 24 °C. All tanks were equipped with recirculating water systems, with 20% of the water replaced weekly. Abiotic parameters, including nitrate and phosphate levels, were monitored bi-weekly. Each tank was fitted with biological filtration systems and artificial seagrass structures to provide shelter and reduce stress. *H. abdominalis* and *S. typhle* were fed daily with live mysid shrimp, *N. ophidion* with live Artemia spp., and *H. formosa* also with the latter supplemented by occasional dried fish flakes. Any uneaten food was removed by suction 30 min after feeding to minimise biofilm development and maintain water quality.

No experimental treatments were applied to the animals before sampling, animals were not allowed to access the opposite sex to avoid any hormonal shifts due to potential priming for mating. We are confident that none of the fish had been recently gravid, because both pipefish species (*Syngnathus typhle* and *Nerophis ophidion*) were collected before the onset of the mating season, and the other two species had been housed sex-separately for several months prior to sampling. Individuals were removed from their aquaria and euthanised in a separate container using an overdose of MS-222 (tricaine methanesulfonate) at a concentration of 500 mg/L. Following euthanasia, fish were weighed, measured (**Supplementary Table S1**) and dissected to collect tissues. From all species, the liver was extracted. In *Hippocampus abdominalis, Syngnathus typhle*, and *Nerophis ophidion*, additional tissues were collected, including the gonads and, in males, also the brood pouch (**Table 1**). *N. ophidion* testes were too small to be processed for hormonal extractions (**Table 1**), and only a limited number of brood pouch samples were available for *H. abdominalis* due to sampling constraints and should thus be considered exploratory (**Table 1**). Due to the small body size of *Heterandria formosa* (maximum ~2 cm), tissues other than liver were too small for hormonal extraction. All tissues were transferred into 2 mL Eppendorf tubes, flash-frozen in liquid nitrogen, and stored at –80 °C until further processing. In addition, blank water samples (800 mL) were collected to account for hormone concentrations in the system water and frozen at -20 °C for a day before further processing.

**Table 1:** Comparison map showing availability of tissue per species.

Species	Liver Female	Liver Male	Ovaries	Pouch	Testes
*H. abdominalis*	9	10	9	4	7
*S. typhle*	12	12	12	12	6
*N. ophidion*	12	12	12	12	0
*H. formosa*	7	7	0	0	0

### Hormone extraction from tissues and water

Steroid hormones were extracted from the water samples using C18 solid-phase extraction (SPE) cartridges (CHROMAFIX C18–480 mg, Macherey Nagel, Germany). Prior to sample loading, the cartridges were conditioned with 6 mL of methanol (99,9% purity) at a flow rate of 1 mL/min using a peristaltic pump (Ecoline, ISMATEC), followed by 6 mL of deionised Milli-Q (MQ) water at the same flow rate. Each 800 mL water sample was weighed and then loaded onto the pre-conditioned cartridges using Tygon LMT-55 tubes (purple/purple size, Carl ROTH, Germany) at a flow rate of 10 mL/min (32, 33). Following sample loading, the cartridges were washed with an additional 6 mL of MQ water at a flow rate of 6 mL/min to remove polar contaminants. Excess water was expelled by pushing air through the cartridges using a syringe, and cartridges were then stored at –20 °C until further processing. For elution, cartridges were thawed to room temperature and reattached to the peristaltic pump using the same tube setup. Hormones were eluted into 5 mL low-protein-binding Eppendorf tubes (ClearLine, Biozym Scintific GmbH, A-1200 Wien) with 4 mL of methanol applied at a flow rate of 1 mL/min. The eluate was evaporated on a heating block at 30 °C (NeoMix 7-0920, NeoLab, Germany) until dry. Dried samples were reconstituted in 220 µL of universal buffer similar to (32) (pH 7.0, 0.5 M Tris, 0.15 NaCl, and MQ water), vortexed for 10 seconds, and stored at –80 °C until hormone quantification.

Liver, gonad, and brood pouch tissues were thawed on ice before extraction. The liver is a key organ for the synthesis and metabolism of many steroid hormones, the gonads are themselves sex-specific endocrine organs, while the brood pouch represents a unique reproductive structure central to paternal gestation in our model system. Each sample was weighed (**Supplementary Table S3**) and transferred to a fresh 2 mL low-protein-binding Eppendorf tube. A phosphate-buffered saline (PBS) solution containing 0.05% sodium azide R620, 0.5% Triton X-100, and a protease inhibitor cocktail (P8340-1ML, SIGMA-ALDRICH, Germany), adjusted to pH 7.2 and pre-cooled to 4 °C, was added in a 1:1 ratio to tissue weight (e.g., 0.08 g of tissue received 800 µL PBS buffer) (33, 34). Samples were left on ice for 10 minutes to equilibrate before they were homogenised with a tissue homogeniser (TissueRuptor II, QIAGEN GmbH, Germany), followed by the addition of 1 mL diethyl ether (99,9% purity). Samples were vortexed vigorously and centrifuged at 12,000 g for 5 minutes. The upper (ether) layer was carefully collected and transferred to a new 2 mL low-protein-binding tube. This extraction step was repeated once more to maximise hormone recovery, yielding approximately 2 mL of diethyl ether extract per sample. The pooled ether phase was evaporated under a fume hood at 30 °C until dry. The dried residue was reconstituted in 1.5 mL Tris-HCl–NaCl universal solution and vortexed thoroughly to ensure complete dissolution. The extract was aliquoted into seven 210 µL portions, each designated for the quantification of one of the seven target hormones. Samples were stored at –80 °C until analysis.

### ELISA

Extracted hormone samples were quantified using commercial enzyme-linked immunosorbent assay (ELISA) kits. CORT, P, E2, and T concentrations were measured using kits from Enzo Life Sciences (Executive Boulevard, Farmingdale, NY 11735, USA). KT was measured using an ELISA kit from Biomol GmbH (Kieler Straße 303a, 22525 Hamburg, Germany), for IT we used a kit from ARBOR ASSAYS™ (1514 Eisenhower Place, Ann Arbor, Michigan 48108 USA), and PRL was quantified using a kit from CUSABIO (7505 Fannin St., Ste 610, Room 7, CUBIO Innovation Center, Houston, TX 77054, USA). PRL in particular uses uIU/mL as unit of measurement, where 1 uIU/mL is approximately equal to a concentration of 0.047 ng/mL (35). All assays were performed according to the manufacturer’s protocols using duplicates. Aliquots were assayed with appropriate kit buffer dilutions adjusted based on sample type and kit. ELISA duplicate readings were averaged for each sample, and the mean optical density of the blank was subtracted. Standard curves were constructed using a four-parameter logistic (4-PL) model implemented with the *dr4pl* package (v. 2.0.0). Concentrations (pg mL^-^¹) obtained from the standard curve were adjusted for the assay dilution factor, transformed to ng, and multiplied by the final reconstitution volume (1.5 mL) to estimate the total hormone content of each sample. Hormone levels were normalised to tissue mass and expressed as ng g^-^¹ wet weight.

### Statistical analysis and visualisation

Morphological measurements, including fish length (cm) and weight (g) were compared across species and sexes. Boxplots were generated in R (v 4.5.1). Prior to statistical testing, data distributions were assessed for normality with the Shapiro–Wilk test. As assumptions of normality were not met, non-parametric tests were applied. Overall differences among groups (species and sex) were tested with the Kruskal–Wallis rank sum test. Where significant effects were detected, *post hoc* pairwise comparisons were performed using Dunn’s test with Bonferroni correction for multiple testing (**Supplementary Table S2**: Sheet A). Boxplots of weight and size can be visualised in Supplementary Material **Supplementary Figure S1**.

Water-borne hormone concentrations were measured in tank water for each species. Due to low or undetectable concentrations for some hormones, certain values fell below the detection limit of the assay and were treated as missing values. The results are visualised in **Supplementary Figure S2**.

Principal component analysis (PCA) was performed on hepatic hormone concentration data normalised to tissue weight (**Figure 2**). For comparability across hormones, data were standardised by transformation into z-scores for each hormone across all samples, such that values represent deviations from the overall mean. PCA was conducted using the pcaMethods package (v. 2.1.0) with the “nipals” algorithm, which accommodates missing values.

**Figure 2:**
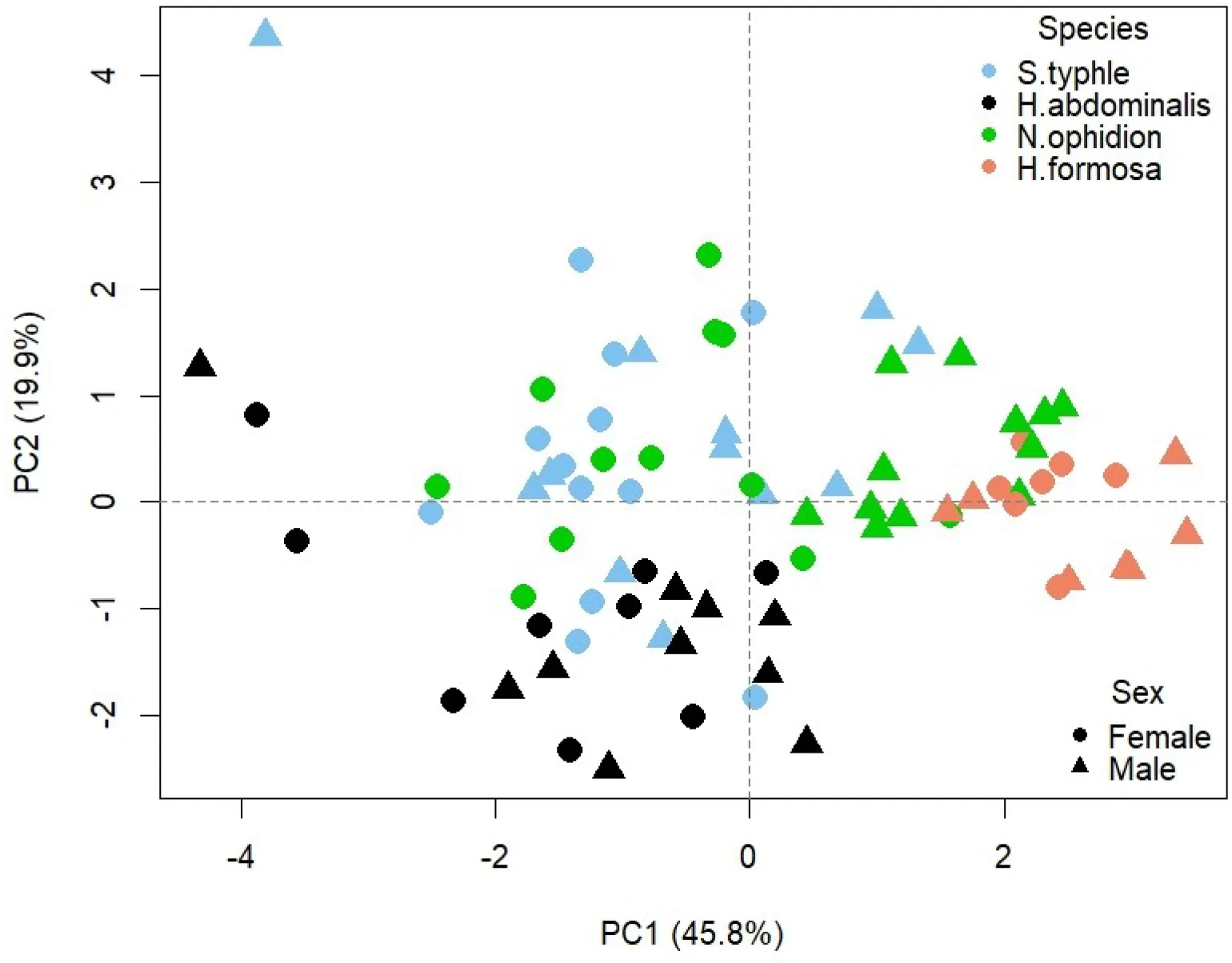
Principal Component Analysis (PCA) of hormonal profiles across species in liver. The PCA is based on seven hormones (z-score normalised for comparability) measured in three syngnathid species and one poeciliid species (black = *Hippocampus abdominalis*; pink = *Heterandria formosa*; green = *Nerophis ophidion*; light blue = *Syngnathus typhle*) across liver, between males (triangle up) and females (triangle down). PC1 explains 45.8% of the total variance and primarily separates *H. formosa* and *N. ophidion* males from the other two species, whereas PC2 explains 19.9% of the variance with *H. abdominalis* clustering separately from all other species.

To further visualise species-specific sex differences in hepatic hormonal profiles, a dot plot representing the magnitude and direction of the sex difference was generated (**Figure 3**). This was calculated as the difference between male and female mean log-transformed z-scores (scale(log10(ng/g)), and significance was tested with either Kruskal-Wallis followed by Dunn test (**Supplementary Table S2**: Sheet D–J).

**Figure 3:**
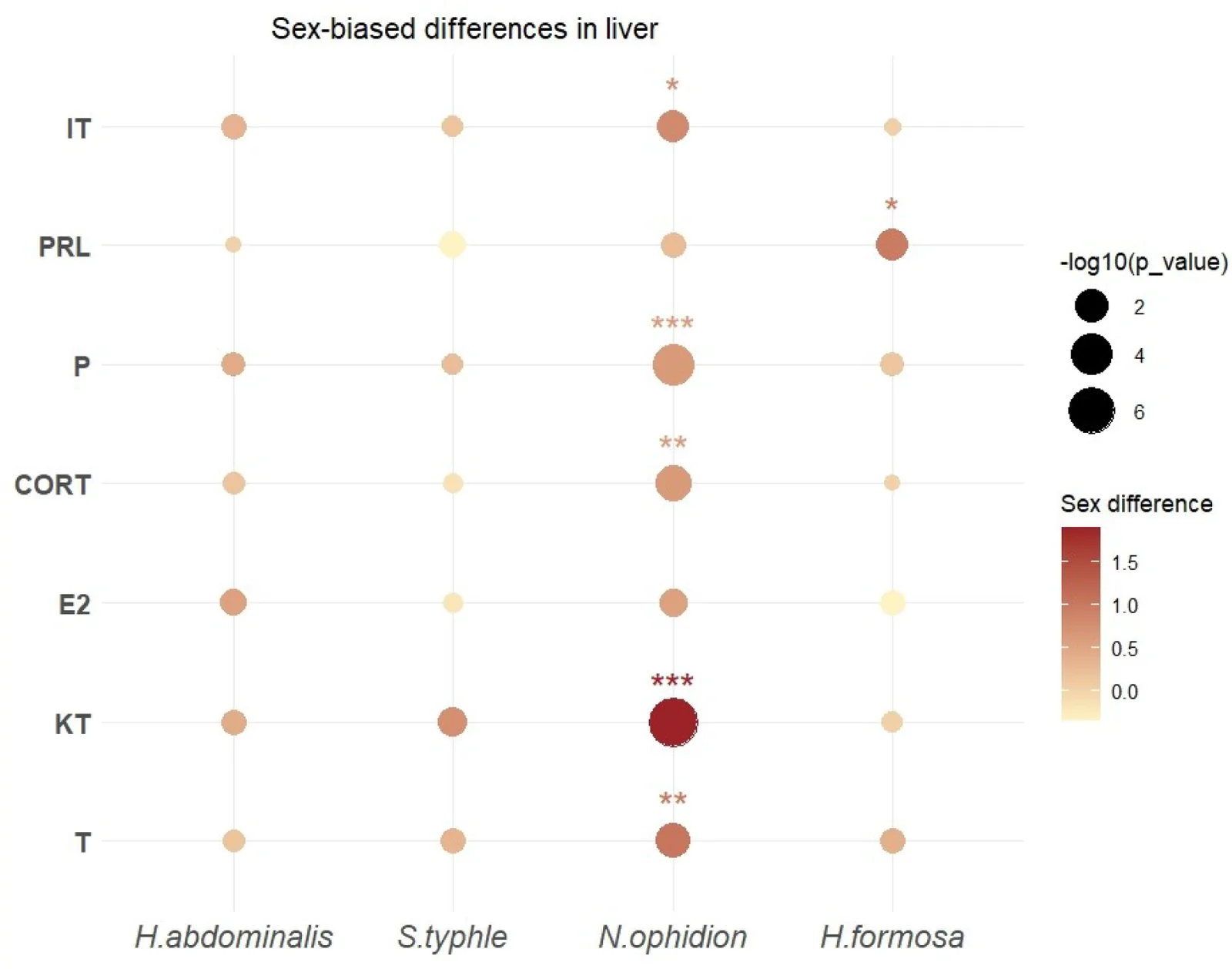
Sex-biased differences in liver hormone levels across species. Dot plot representing sex-biased differences across seven hormones on the y-axis, and the four species on the x-axis. The colour gradient shows the magnitude of the sex divergence, ranging from light yellow (low/no difference) to deep maroon (high difference). The circle size corresponds to the statistical significance as -log10(p-value), where circles indicate higher statistical significance. While the asterisks above individual dots denote statistical significance thresholds (*p < 0.05, **p < 0.01, ***p < 0.001).

Statistical analyses of hormonal concentrations were primarily conducted using linear mixed-effects models (LMMs) with species and combined tissue-sex factor as fixed effects, and plate and individual identity as random effects (**Supplementary Table S2**: Sheet B). This primary framework accounted for the unbalanced experimental design (e.g., tissues unique to specific sexes or species) as well as technical batch effects and repeated measures within individuals. Hormonal concentrations were z-score transformed prior to model fitting. *Post hoc* pairwise comparisons between species were performed using estimated marginal means (*emmeans*) with Tukey adjustment. To evaluate liver tissue, the only tissue shared across all four species, a dedicated cross-species LMM was fitted on liver-specific z-scores (**Supplementary Table S2**: Sheet C). Similarly, syngnathid brood pouches were compared by fitting separate LMMs for each log10-transfomed hormone, with species as a fixed effect and plate as a random effect (**Figure 4**); back-transformed estimated marginal means with 95% confidence intervals were extracted for plotting.

**Figure 4:**
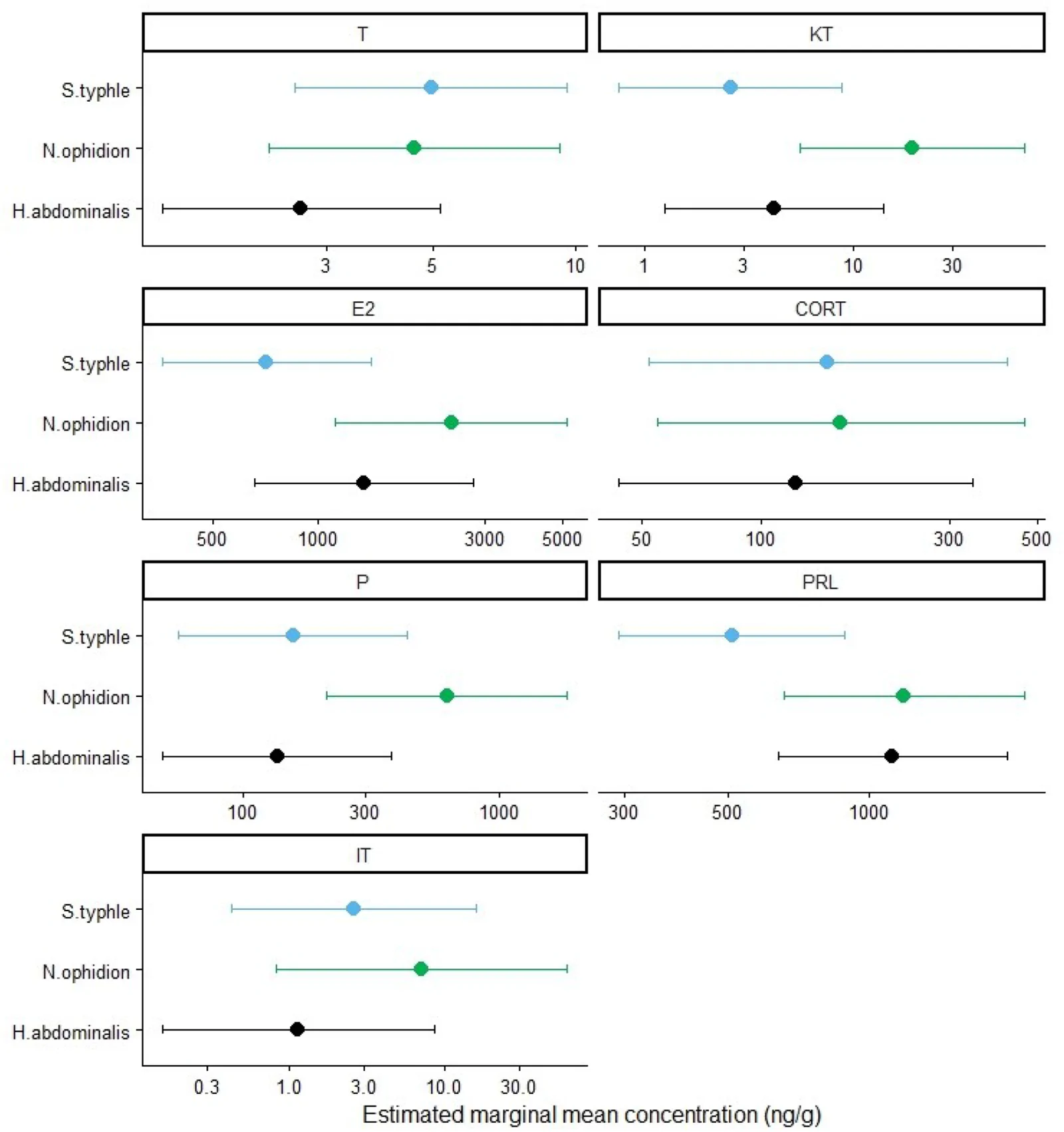
Estimated marginal mean concentrations of hormone concentrations across syngnathid species in pouch. Estimated marginal mean concentrations (ng/g) are shown for *S. typhle* (blue*), N. ophidion* (green), and *H. abdominalis* (black) across the hormone groups T, KT, E2, CORT, P, PRL, and IT. Points represent estimated marginal means, and horizontal error bars indicate the corresponding uncertainty intervals (e.g., 95% confidence intervals). The x-axis is presented on a logarithmic scale, with scale limits and tick marks varying among panels to accommodate differences in compound concentrations.

To evaluate specific single-variable or tissue-isolated patterns, supplementary analyses were conducted separately for each hormone (**Supplementary Table S2**: Sheets D–J). For these subset datasets, data normality was checked via Shapiro-Wilk tests; normally distributed groups were compared using one-way ANOVA with Tukey’s *post hoc* tests, whereas non-normal datasets were evaluated using Kruskal-Wallis rank sum tests followed by Dunn’s *post hoc* tests with Benjamini-Hochberg correction. Additionally, gonadal hormone ratios between E2, KT, and T were calculated using two-way ANOVA with species, sex, and their interaction (**Figure 5**; **Supplementary Table S2**: Sheet K).

**Figure 5:**
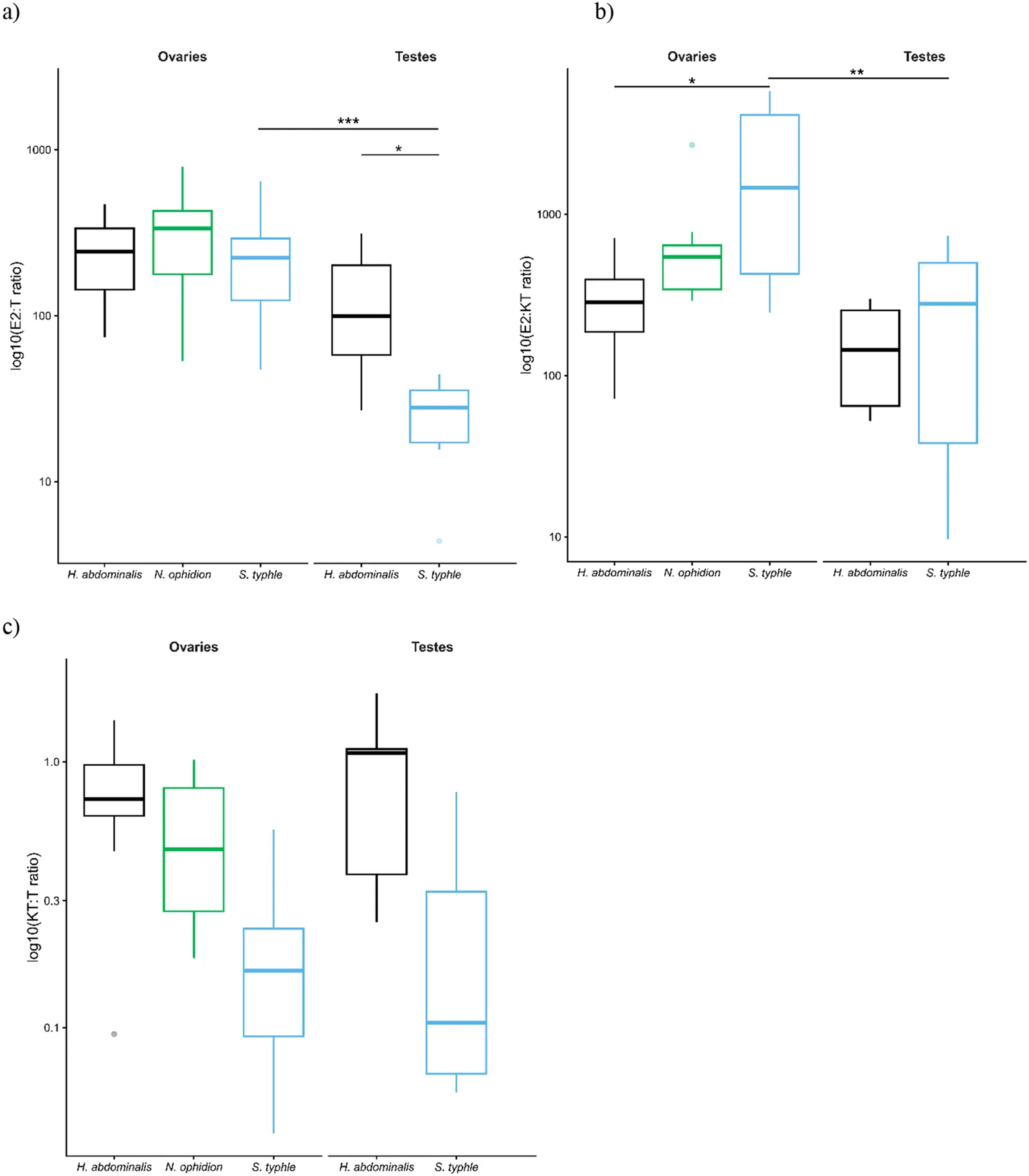
Gonad sex-hormone ratios across syngnathids. Boxplots show the distribution of the log-transformed ratios a) estradiol-to- testosterone, b) estradiol-to-11-Keto-Testosterone, c) 11-Keto-Testosterone-to-Testosterone, in ovaries and testes of *H. abdominalis* in black, *N. ophidion* in green, and *S. typhle* in blue. The y-axis is presented on a logarithmic scale. Only species with available samples for each tissue are shown. While the asterisks above individual dots denote statistical significance thresholds (*p < 0.05, **p < 0.01, ***p < 0.001).

Finally, species-specific Spearman’s rank correlations were used to assess relationships between hepatic hormone concentrations and individual body weight, as well as between hormone concentrations and reproductive investment (gonadosomatic index, GSI; calculated as ovary mass divided by total body mass) in *H. abdominalis*, *S. typhle*, and *N. ophidion* (**Supplementary Figure S3**; **Supplementary Table S2**: Sheets D–J).

## Results

### Endocrine divergence is driven by species in hepatic tissue

The hormonal liver profiles revealed clear separation among species, sex (LMM, p < 0.0001), and their interaction (LMM, p < 0.01; **Supplementary Table S2**: Sheet C). In the PCA from **Figure 2** we find that PC1 (45.8%) separates *H. formosa* and *N. ophidion* males, specifically, from the rest. In fact, we find the most significant difference between *H. formosa* and all other species (EMM, adj.p < 0.001). Interestingly, killifish females were more similar to the straightnosed pipefish males (EMM, adj.p > 0.05) compared to the male killifish (EMM: adj.p < 0.01). Example, some of the hormonal concentrations in the liver of *H. formosa* were similar to *N. ophidion* male concentrations, such as T (23.8 ± 4.64 ng/g in male and 14.95 ± 1.99 ng/g in female *H. formosa*, vs. 13.82 ± 1.86 ng/g male and 7.15 ± 2.37 ng/g in female *N. ophidion*; **Supplementary Figure S4A**), IT (74.42 ± 26.44 ng/g in male and 60.71 ± 20.8 ng/g in female *H. formosa*, vs. 64.91 ± 17.76 ng/g and 17.19 ± 4.46 ng/g; **Supplementary Figure S4G**), and P (843.67 ± 84.17 ng/g in male and 742.84 ± 33.30 ng/g in female *H. formosa*, vs. 601 ± 31.06 ng/g and 393.97 ± 33.06 ng/g; **Supplementary Figure S4E**). These were on average significantly higher than in the other syngnathid species (EMM p < 0.001; **Supplementary Table S2**: Sheet C). Also, CORT levels were disproportionately higher in *H. formosa* (612.75 ± 74.56 ng/g males; 642.82 ± 124.90 ng/g females), compared to syngnathids liver (**Supplementary Figure S4D**). The latter were rather uniform, displaying no pronounced syngnathid-wide differences (EMM, p > 0.05, **Supplementary Table S2**: Sheet G). Concentrations averaged around the 80–180 ng/g mark (**Supplementary Figure S4D**), with only sex-specific variations in *N. ophidion* (178.22 ± 27.58 ng/g males; 94.43 ± 8.16 ng/g females; **Supplementary Figure S4D**). We found a significant positive correlation between hepatic CORT concentration and body weight for both *S. typhle* (r = 0.76, p < 0.0001) and *N. ophidion* (r = 0.57, p < 0.01). While, E2 liver concentrations, for example, were overall relatively similar across all species (**Supplementary Figure S4C**; LMM p > 0.05; **Supplementary Table S2**: Sheet F).

PC2 (19.9%) appears to distinguish between *H. abdominalis* and the other species (**Figure 2**), it did not reach statistical significance (**Supplementary Table S2**: Sheet C). This difference was mostly driven by PRL, as it had its highest concentration in the seahorse’s liver (**Supplementary Figure S4F**; **Supplementary Table S2**: Sheet I), with male liver concentrations averaging 3037.64 ± 1026.1 ng/g and female liver concentrations 2498.42 ± 811.78 ng/g. However, these elevated mean values were accompanied by strong inter-individual variation, with a few individuals substantially influencing the overall distribution (**Supplementary Figure S4F**). Notably, two males (2M in **Supplementary Table S1**: 25.5 cm, 30.5 g; liver: 8904.52 ng/g; testes: 1653.32 ng/g; and 3M in **Supplementary Table S1**: 24.4 cm, 21.54 g; liver: 8694.43 ng/g; testes: 592.61 ng/g) and one female (2F in **Supplementary Table S1**: 27 cm, 24.8 g, GSI 1.5; liver: 8123.69 ng/g; ovaries: 3915.54 ng/g) exhibited exceptionally high PRL levels. Conversely, one male (1M in **Supplementary Table S1**: 22.5 cm, 18.04 g; liver: 152.38 ng/g; testes: 190.32 ng/g) and one female (1F in **Supplementary Table S1**: 25.5 cm, 40.24 g, GSI 2.8; liver: 223.12 ng/g; ovaries: 311.12 ng/g) showed comparatively low concentrations. These extreme PRL values were observed in larger individuals and contributed to high inter-individual variation. Indeed, hepatic PRL concentrations correlated positively with body mass in *H. abdominalis* (r = 0.48, p = 0.04). Additionally, the detected levels may reflect a combination of systemic circulation and local sequestration, making it difficult to definitively confirm tissue-specific steroid regulation. For the other two syngnathid species we found similar concentrations in both sexes (*S. typhle:* 665.43 ± 109.06 ng/g female; 507.24 ± 81.2 ng/g male; *N. ophidion*: 642.62 ± 130.77 ng/g females; 712.25 ± 60.87 ng/g males). Although, we did also identify a significant positive correlation between hepatic PRL concentrations and body mass in *S. typhle* (r = 0.82, p < 0.0001).

Within species hepatic sex separation was mostly apparent for *N. ophidion* (EMM, adj.p < 0.0001, **Supplementary Table S2**: Sheet C). This pattern is evident in the follow-up dot plot (**Figure 3**), which reveals species-specific sexual heteromorphism across the four species in the liver tissue. *N. ophidion* shows widespread male-biased hormonal differences, particularly for androgens (KT in males 8.37 ± 1.78 ng/g vs. in females 1.67 ± 0.16 ng/g, **Supplementary Figure S4B**; T in males 13.82 ± 1.86 ng/g vs. in females 7.15 ± 0.76 ng/g; **Supplementary Figure S4A**), P (males 601 ± 31.06 ng/g vs. females 393.97 ± 33.06 ng/g; **Supplementary Figure S4E**), CORT (males 178.22 ± 27.58 ng/g vs. females 94.43 ± 8.16 ng/g; **Supplementary Figure S4D**), and IT (males 64.91 ± 17.76 ng/g vs. females 17.19 ± 4.46 ng/g; **Supplementary Figure S4G**). Only *H. formosa* showed a strong male-biased concentration of PRL (1990.77 ± 574.81 ng/g in males; 804.82 ± 136.81 ng/g in females; Kruskal p = 0.013; **Supplementary Table S2**: Sheet I; **Supplementary Figure S4F**).

Noteworthy, hepatic IT concentrations were generally lower in females than males across species (**Supplementary Figure S4G**), with *H. formosa* females averaging 60.71 ± 20.8 ng/g compared to 74.42 ± 26.44 ng/g in males; *N. ophidion* females 17.19 ± 4.46 ng/g compared to 64.91 ± 17.76 ng/g in males (Dunn-Test adj. p = 0.02); and *S. typhle* females 8.33 ± 4.30 ng/g compared to 12.13 ± 5.18 ng/g in males. In *H. abdominalis*, hepatic IT concentrations were similar between females (3.35 ± 2.4 ng/g) and males (2.79 ± 0.62 ng/g) (**Supplementary Table S2**: Sheet J). Comparisons between sex-specific species hepatic concentrations further showed that female *H. formosa* differed significantly from all other syngnathid females (EMM adj. p < 0.05, **Supplementary Table S2**: Sheet J). Among males, there was a strong significant difference between *H. abdominalis* and both *H. formosa* and *N. ophidion* (EMM adj. p < 0.0001), but not *S. typhle* (**Supplementary Table S2**: Sheet J), while *S. typhle* males also differed significantly from *H. formosa* and *N. ophidion* males (EMM adj. p < 0.05).

### Pouch-specific steroid enrichment in *N. ophidion* elevated compared to other Syngnathids

When examining individual hormones in pouch tissue, *N. ophidion* exhibited higher concentrations of KT (18.72 ± 2.19 ng/g) compared to *S. typhle* (3.32 ± 1.01 ng/g) and *H. abdominalis* (4.48 ± 0.72 ng/g; EMM adj. p < 0.001, **Figure 4**; **Supplementary Figure S4B**). A similar pattern was observed for P, with *N. ophidion* showing markedly elevated levels (1020.78 ± 84 ng/g) relative to *S. typhle* (227.77 ± 60.44 ng/g) and *H. abdominalis* (209.23 ± 75.42 ng/g) (EMM adj. p < 0.002, **Figure 4**; **Supplementary Figure S4E**). E2 was also significantly higher in *N. ophidion* pouch tissue (2827.03 ± 497.11 ng/g) compared to *S. typhle* (833.23 ± 112.51 ng/g; EMM adj. p = 0.0003), although levels in *H. abdominalis* (1610.27 ± 197.07 ng/g) were not significantly different (**Figure 4**; **Supplementary Figure S4C**). However, pouch IT concentrations were comparable between *N. ophidion* (14.2 ± 3.35 ng/g) and *S. typhle* (11.43 ± 6.59 ng/g), but somewhat lower in *H. abdominalis* (2.97 ± 2.09 ng/g) despite not being significantly different (**Figure 4**; **Supplementary Figure S4G**; **Supplementary Table S2**: Sheet J). While, PRL concentrations were elevated in both *H. abdominalis* (1207.96 ± 147.91 ng/g) and *N. ophidion* (1224.45 ± 141.11 ng/g), showing similarly high levels between these two species, compared to *S. typhle* (550.39 ± 69.27 ng/g) (**Figure 4**; **Supplementary Figure S4F**). CORT was, like in liver, consistently similar across syngnathid pouch tissue (**Figure 4**), with *S. typhle* showing 227.77 ± 60.44 ng/g, *N. ophidion* 152.12 ± 17.91 ng/g, and *H. abdominalis* 133.05 ± 17.36 ng/g (**Supplementary Figure S4D**).

### Gonadal hormone profiles reveal distinct sex- and species-specific patterns

The gonadal tissue revealed that sex-steroid hormones (T, KT, E2) were significantly different between males and females (Dunn test adj. p ≤ 0.05; **Supplementary Table S2**: Sheet D–F). In particular, T concentrations were highest in *S. typhle* testes (25.35 ± 3.7 ng/g). While, ovarian E2 concentrations were generally high and comparable across syngnathid, including *S. typhle* (1428.62 ± 241.88 ng/g), *N. ophidion* (1112.96 ± 142.7 ng/g), and *H. abdominalis* (1183.6 ± 237.76 ng/g), with no significant differences among these groups (**Supplementary Figure S4C**; **Supplementary Table S2**: Sheet F). In contrast, E2 levels were significantly lower in *H. abdominalis* testes (499.04 ± 130.69 ng/g), as well as in *S. typhle* testes (646.62 ± 154.41 ng/g) (Dunn test adj. p < 0.05; **Supplementary Table S2**: Sheet F). When comparing specific sex-hormone ratios within gonads we found that both the E2:T and E2:KT ratios differed markedly between ovarian and testicular tissues (ANOVA, *p* < 0.0001; **Supplementary Table S2**: Sheet K), with ovaries generally exhibiting a more estrogen-dominated steroid profile and testes a more androgen-dominated profile (**Figures 5a, b**). However, species-specific differences were also evident. For the E2:T ratio, *S. typhle* exhibited a significantly lower ratio in testes than *H. abdominalis* (EMM, adjusted *p* = 0.02; **Supplementary Table S2**: Sheet K), whereas the E2:KT ratio did not differ significantly between these species in testes. In ovaries, the E2:T ratio was broadly similar among the three syngnathid species (**Figure 5a**). On the contrary, the E2:KT ratio was markedly higher in *S. typhle* ovaries than in *H. abdominalis* and *N. ophidion*, although the difference was statistically significant only relative to *H. abdominalis* (EMM, adjusted *p* = 0.02; **Figure 5b**). Interestingly, the KT:T ratio showed a statistically consistent relationship between the two androgens across tissues within species (**Figure 5c**). Neither *S. typhle* nor *H. abdominalis* showed a significant difference in the KT:T ratio between ovaries and testes (**Supplementary Table S2**: Sheet K). Although *H. abdominalis* appeared to have a slightly higher KT:T ratio than the other two species, indicating a relatively greater contribution of KT to the androgen profile, these differences were not statistically significant.

We also found that ovarian GSI had some influence on hormone levels across species. In *N. ophidion*, ovarian E2 GSI was positively correlated with total ovarian hormone concentration (ng) (r = 0.71, p = 0.013). This pattern was also observed for T in *N. ophidion* ovaries (r = 0.75, p < 0.01). For KT, GSI was strongly positively correlated with ovarian concentration in *N. ophidion* (r = 0.94, p < 0.0001), whereas a weak negative relationship was observed in *S. typhle* ovaries (r = -0.064, p = 0.03).

In contrast, ovarian P concentrations were relatively similar across species, with values ranging from 142.63 ± 23.78 ng/g in *S. typhle* to 381.07 ± 66.65 ng/g in *N. ophidion* and intermediate levels in *H. abdominalis* (175.7 ± 39.55 ng/g), and also no differences based on GSI. Similarly for IT concentrations, which did not differ significantly among species or between ovarian and testicular tissue (**Supplementary Figure S4G**; **Supplementary Table S2**: Sheet J). Although concentrations varied somewhat numerically among groups with mean values ranging from 3.57 ± 1.78 ng/g in *H. abdominalis* ovaries to 19.51 ± 10.51 ng/g in *S. typhle* testes. *N. ophidion* ovaries (15.29 ± 6.28 ng/g) and *S. typhle* testes showed the highest mean IT concentrations, whereas concentrations were lower in *H. abdominalis* ovaries and testes (3.57 ± 1.78 and 4.20 ± 1.56 ng/g, respectively) and *S. typhle* ovaries (5.47 ± 2.29 ng/g).

CORT concentrations were relatively similar between male and female gonadal tissues within species (**Supplementary Figure S4D**; **Supplementary Table S2**: Sheet G), but *S. typhle* did have slightly higher levels (ovaries 142.63 ± 23.78 ng/g; testes 161.06 ± 28.59 ng/g) compared to *H. abdominalis* (Ovaries 65.36 ± 6.66 ng/g; testes 82.25 ± 9.61 ng/g) (Dunn test adj. p ≤ 0.05). Correlations between GSI and ovarian CORT concentrations revealed a negative correlation for *S. typhle* (r = -0.71, p = 0.02).

PRL concentrations showed evidence of tissue- and species-specific variation (**Supplementary Table S2**: Sheet I). In *H. abdominalis*, ovarian PRL concentrations were higher than those measured in testes (1535.52 ± 407.09 and 650.94 ± 174.86 ng/g, respectively; Dunn test, adjusted *p* = 0.05). Instead, PRL concentrations in *S. typhle* were similar between ovaries and testes (701.08 ± 113.72 and 658.31 ± 199.39 ng/g, respectively), while ovarian concentrations in *N. ophidion* were comparable to those observed in *S. typhle* (**Supplementary Figure S4F**). Ovarian PRL concentrations in *H. abdominalis* were also higher than those in *S. typhle* and *N. ophidion* ovaries, although these differences were marginally significant (Dunn test, adjusted *p* = 0.05; **Supplementary Table S2**: Sheet I). Despite these differences, PRL considerable variation in PRL concentrations was observed among individual samples within species and tissue types.

## Discussion

Hormonal systems both shape and respond to variation in reproductive strategies, including mating behaviours, and parental care. Nonetheless, most current research is heavily biased towards species with conventional reproductive strategies, where biological sex and reproductive roles are tightly coupled. This narrow focus limits our ability to understand how endocrine regulation is organised in taxa where these reproductive roles and parental care strategies deviate from the classical vertebrate pattern. By comparing concentrations of seven hormones across multiple tissues in species representing a gradient of parental investment and reproductive strategies, we hypothesised that endocrine profiles are not fixed to biological sex alone, but covary with variation in life-history strategies, including variation in reproductive roles and parental care.

### Major hormonal architecture divergence between Poeciliids and Syngnathids

The comparison of hepatic hormonal profiles across the four species revealed a phylogenetic separation between *H. formosa* and the three syngnathid species (**Figure 2**, EMM, adj. p < 0.001). *H. formosa*, a species with a high matrotrophy index (30, 36), exhibited a distinct endocrine signature characterised by higher baseline levels of CORT, T, P, and IT, whereas E2, KT, and PRL remained more comparable to syngnathid species. While cross-species comparisons of absolute hormone concentrations using immunoassays should be interpreted with caution due to potential differences in antibody affinity, matrix effects, tissue composition, and extraction efficiency, as well as the inability of whole-tissue homogenates to distinguish local synthesis from systemic circulation or tissue sequestration, the pattern of divergence is selective rather than uniform across all measured hormones, suggesting that it is unlikely to be explained by methodological bias alone.

The strongest differences were for CORT (**Supplementary Figure S4D**), being significantly higher in *H. formosa* compared to the syngnathid species (EMM p.adj. < 0.0001). CORT is a primary glucocorticoid involved in regulating stress responses and energy allocation in teleosts metabolism (37, 38). Baseline CORT levels can vary substantially among species due to differences in the regulation of the hypothalamic–pituitary–interrenal (HPI) axis (39). Comparative studies between live-bearing and lecithotrophic fish species have also reported elevated CORT levels in *H. formosa*, which have been interpreted as reflecting higher energetic costs associated with a preparatory endocrine state supporting reproduction (36).

Beyond reproduction, behavioural and ecological differences between taxa may contribute to these patterns. Poeciliids are typically more active swimmers, whereas syngnathids generally exhibit slower, more sedentary locomotion. Increased locomotor activity has been associated with elevated metabolic demand and corresponding increases in CORT levels in fish (40). Nonetheless, linking swimming speed directly to CORT variation would require integrating molecular data, for example, through gene expression of components of the stress and reproductive axes. In contrast to steroid hormones, we also found that PRL had its highest concentration in *H. abdominalis* liver (**Supplementary Figure S4F**), likely driving in part the separation with other species in PC2 (**Figure 2**). In teleosts, PRL is produced by the pituitary gland and can be locally regulated, with potential accumulation in tissues depending on binding or retention dynamics (41). It is a highly pleiotropic hormone with more than 300 known activities (18). While it is best known for its role in osmoregulation, particularly in maintaining ion balance in freshwater environments (41), it also contributes to reproductive processes, including parental care (18), where it may regulate epithelial and ion transport processes in brood pouch tissues (42). While concentrations were highest in *H. abdominalis* (**Supplementary Figure S4F**), a species characterised by high paternal investment (19, 43), substantial inter-individual variation was observed. We also found elevated concentrations in pouch tissue of *N. ophidion*, a species lacking a sealed brood pouch, but moderate levels in *S. typhle* (**Figure 4**). Thus, PRL concentrations did not scale consistently with potential parental investment, indicating that PRL variation cannot be explained solely by reproductive strategy. Importantly, all individuals were sampled outside of active brooding, suggesting that PRL values may not reflect the time in which parental care is actively provided. Instead, these findings suggest that PRL reflects a combination of tissue-specific functions and physiological state. The pronounced variability observed is consistent with previous literature highlighting that PRL cannot be generalised to all teleost species (41), with marked interspecific differences in its physiological roles, particularly in relation to osmoregulation (44). This is of particular importance considering that these species also differ in their salinity environment (freshwater, brackish, and marine), which is likely to influence PRL’s osmoregulatory roles.

### Endocrine sexual heteromorphism aligns with reproductive role and investment

Across the studied species, the degree of sexual heteromorphism in hormonal profiles broadly reflects differences in mating systems and reproductive biology. The syngnathid species included in this study also exhibit considerable diversity in sex determination systems, ranging from XY systems in *Hippocampinae* to sex determination potentially involving single genes or environmental influences in *Syngnathidae* and *Nerophinae* (28). This variation adds an additional layer of biological complexity that may contribute to interspecific differences in endocrine regulation, as variation in sex determination pathways can be linked to differences in downstream hormonal sensitivity, including receptor expression and steroidogenic enzyme activity.

The strongest hormonal sexual heteromorphism was observed in *N. ophidion*, where male and female liver tissues differed significantly (**Figure 3**; EMM adj. p < 0.001, **Supplementary Table S2**: Sheet C), potentially relating to its polyandrous mating system (45, 46). In *S. typhle*, a polygynandrous species (29, 47), sex differences were less pronounced, with no significant overall differences in liver tissue, although gonadal tissues revealed clearer divergence in individual hormones. Here, androgens (T and KT) were elevated in testes, while E2 was higher in ovaries (Dunn test p < 0.01, **Supplementary Table S2**: Sheet D–F, J), consistent with established sex steroid patterns in teleosts and previous findings in pipefish (26).

In contrast, both *H. abdominalis* and *H. formosa* exhibited limited sexual heteromorphism in their hepatic hormonal profiles (**Figure 3**; **Supplementary Table S2**: Sheet B–C), and somewhat decreased also in the gonadal tissue of *H. abdominalis* (**Figure 5**). This pattern is notable given the divergent reproductive roles of the sexes in these species, including male pregnancy in *H. abdominalis* versus female pregnancy in *H. formosa*, coupled by rather conventional reproductive roles. Nonetheless, because individuals were not sampled during pregnancy or courtship, the present data do not allow us to determine whether reproductive state contributes to the observed patterns of sexual heteromorphism. The comparatively similarly low levels may instead reflect species-specific characteristics of endocrine regulation, although this remains to be tested across different reproductive states. In *H. formosa*, hormone measurements were limited to liver tissue, which primarily reflects hormone metabolism and may therefore not capture sex-specific differences occurring in reproductive tissues (15).

### Specialised brood pouches exhibit reduced steroidogenic signatures

The evolution of male pregnancy in syngnathids provides a gradient of brooding complexity that enables comparative investigation of associated endocrine patterns. In our dataset, ventral skin tissue from *N. ophidion* differed markedly from the brood pouch tissues of both *S. typhle* and *H. abdominalis* (**Figure 4**, LMM adj. p < 0.001), whereas no significant differences were detected between the latter two species for the hormonal brood pouch architecture (**Supplementary Table S2**: Sheet B). This could suggest that the presence of a specialised brood pouch, rather than its degree of complexity, is associated with distinct endocrine characteristics. Given that individuals were not pregnant, these patterns likely reflect the baseline physiological state of the tissues.

For individual hormones in pouch tissue, *N. ophidion* exhibited substantially higher concentrations of P, KT, and E2 compared to both pouch-bearing species, with differences of approximately 3–5-fold for KT and P and more than twofold for E2 (**Figure 4**). Rather than demonstrating pouch-specific functional requirements, because the male “pouch” in *N. ophidion* consist of relatively unmodified ventral skin, this tissue may simply retain a default steroid-sensitive state typical of the body wall, seeing as similar levels were also observed in liver tissue. Additionally, phylogenetic relatedness may also contribute to the observed differences in hormonal profiles. *Syngnathus* and *Hippocampus* are sister genera, whereas *Nerophis* belongs to a more distantly related trunk-brooding lineage. Brooding structures have evolved independently across these lineages, and the regulatory mechanisms underlying their development maybe unique. Thus, differences in hormonal profiles between these groups should also be interpreted in light of their distinct evolutionary histories.

It is worth noting that E2 concentrations were comparatively higher in the pouch tissue of *H. abdominalis* than in *S. typhle* (**Figure 4**). Estrogens are known to influence vascularisation, epithelial remodelling, and inflammation modulation (48), processes that may be particularly important in the highly vascularised and spongy brood pouch tissue of *H. abdominalis*. Even so, literature in *Syngnathus scovelli*, showed that exposure to synthetic estrogen affected brood-pouch development, while also inducing female-typical ornamentation (49–51). Furthermore, the elevated concentrations of steroid hormones in the ventral tissue of *N. ophidion* compared with the more specialised brood pouch tissue suggest that tissue-specific steroid profiles may reflect species-specific characteristics of reproductive tissues rather than brooding structure alone. Sex steroids, including P, KT and E2, can act as developmental regulators in fishes and influence processes such as growth and sex differentiation (52–54), and the more complex brood pouches of *S. typhle* and *H. abdominalis* may require a more tightly regulated endocrine environment to accommodate for future embryo incubation. Nonetheless, because the sampled males were non-pregnant, the present data cannot distinguish whether local steroid levels reflect baseline suppression relevant to brood-pouch and/or embryo development, or an inactive state prior to pregnancy-induced upregulation. Moreover, exceptionally high absolute levels should be interpreted with caution. This is particularly relevant for the E2 results, given the 4.64% cross-reactivity of the ELISA kit with estrone (E1); the much higher E2 concentrations in *N. ophidion* pouch tissue could partially reflect signal inflation from even minor cross-reactivity with E1.

## Conclusion

The pleiotropic role of hormones in regulating sexual traits, sex-biased gene expression, and reproductive behaviour is well established (55). Nonetheless, the extent to which vertebrate endocrine systems are evolutionarily conserved versus flexible remains debated (55, 56). Our comparative analysis of baseline hormonal set-points shows that hormonal architecture is likely shaped not only by biological sex, but also by evolutionary history and species-specific life-history strategies. The strong divergence between the matrotrophic poeciliid *H. formosa* and the syngnathids could primarily reflect phylogenetic distance, but also differences in reproductive mode, energetic demands, and ecology. Within syngnathids, the evolution of specialised brood pouches was associated with reduced steroidogenic signatures, suggesting tighter endocrine regulation of brooding tissues to support pouch tissue. Endocrine sexual heteromorphismalso broadly aligned with mating system and parental investment, with the strongest sex-biased hormonal regulation in *Nerophis ophidion* and reduced differentiation in species with greater paternal investment and lower mating competition. Together, these findings suggest that shifts in reproductive roles and parental investment could coincide with corresponding changes in endocrine architecture, highlighting both the evolutionary flexibility and functional constraints of hormonal systems.

Nevertheless, because brood pouch complexity and sex-role reversal co-vary across our sampled syngnathids as a combined axis of derived parental investment, our current study design cannot separate their relative contributions. Disentangling whether pouch morphology or mating system acts as the primary driver would require a broader phylogenetic comparison incorporating species with rarer trait combinations (such as polyandrous species possessing highly complex pouches). Future studies expanding this taxonomic coverage will be essential to map these distinct evolutionary trajectories, further clarifying both the evolutionary flexibility and functional constraints of vertebrate hormonal systems.

## Data Availability

Further data and information can be found in the **Supplementary Material**. **Supplementary Data Sheet 1** contains **Supplementary S1** to **S3**. **Supplementary Figure S1** are boxplots of weight and length of the fish species used; **Supplementary Figure S2** shows the seven different hormone concentrations in the species-specific tanks; **Supplementary Figure S3** has the Gonadosomatic index values of the syngnathid species; **Supplementary Figure S4** individual hormonal concentrations across all species and tissue types. **Supplementary Table S1** contains weight and length of all the sampled individuals. **Supplementary Table S2** contains the results of the statistical analysis and is divided in in 10 sheets; Sheet A has the statistics for the morphology data; Sheet B has the statistics on the seven hormones across species and tissues in z-scores; Sheet C contains a liver specific statistical analysis across the four species and seven hormones; Sheet D to J have hormone specific statistics including correlation analysis – Sheet D for Testosterone, Sheet E for 11-Keto-Testosterone, Sheet F for Estradiol, Sheet G for Cortisol, Sheet H for Progesterone, Sheet I for Prolactin, Sheet J for Isotocin, and Sheet K for the gonadal ratios. **Supplementary Table S3** contains the raw data of the ELISA results for each hormone.
