# Supplementary material for: Sex-specific hormone signatures in four fish species with divergent reproductive strategies
Source: Front Endocrinol (Lausanne). 2026 Oct 2;17:1923972. doi: 10.3389/fendo.2026.1923972 (PMC13672188; doi:10.3389/fendo.2026.1923972)
Supplement: Supplementary file 1 [file SupplementaryFile1.docx]

Supplementary Material

Sex-specific steroid hormones signatures in four fish species with divergent reproductive strategies.

Results

**Figure S1:** Boxplot comparison of fish weight (a) and length (b) across four fish species, separated by sex. The x-axis shows the four species, while boxplot outline colours indicate sex (male vs. female). Length is shown in centimetres (cm) and weight in grams (g). Boxes represent the interquartile range (IQR), horizontal lines the median, whiskers the data range excluding outliers.

The fish species differed highly significantly in overall body morphology (Table S2, Sheet A). The strongest divergence was observed between *Heterandria formosa*, the smallest species, and *Hippocampus abdominalis*, the largest. In *H. formosa*, females (n = 7) had a mean body mass of 0.09 ± 0.009 g and a mean length of 2.0 ± 0.05 cm, whereas males (n = 8) weighed 0.035 ± 0.005 g and measured 1.7 ± 0.05 cm. In contrast, *H. abdominalis* females (n = 9) had a mean body mass of 27.6 ± 3.7 g and a mean length of 25.5 ± 1.0 cm, while males (n = 10) weighed 20.4 ± 2.6 g and measured 23.2 ± 1.3 cm. In *Syngnathus typhle*, females (n = 12; 1.63 ± 0.44 g; 16.1 ± 1.4 cm) and males (n = 12; 1.20 ± 0.33 g; 14.9 ± 1.4 cm) were similar in both body mass and length. Overall, no species exhibited significant sexual dimorphism in body mass. Sexual dimorphism in length was detected only in *Nerophis ophidion*, where females (n = 12; 24.3 ± 0.66 cm) were significantly longer than males (n = 12; 17.0 ± 0.72 cm; Dunn’s test, p = 0.02).

**Figure S2.** Concentrations of seven hormones measured in species-specific tank water. Points represent species-specific water hormone concentrations that were normalised for water volume (ng g⁻¹). Hormones are shown on the x-axis, and colours indicate species identity. Some values are missing because hormone concentrations were below the detection limit of the assay.

As shown in Figure S2, water-borne hormone concentrations were generally very low across species-specific tanks, with values ranging from 0.01 ng/L to 3 ng/L. Several values were below the detection limit of the assay. For example, prolactin (PRL) was only detectable in *H. formosa*. PRL has a different chemical structure to steroid hormones, since it is a large peptide relying on its tertiary protein structure (Manzon, 2002), which can be structurally fragile in saline environments since high salinity can in some proteins promote aggregation and/or make it more susceptible to enzymatic breakdown (Kohn et al., 1997), ultimately reducing the amount of biologically active hormone in saline environments. Furthermore, *H. abdominalis* tank water contained detectable levels only for estradiol (E2) and progesterone (P). This could be in part due to the higher salinity in the tank water of *H. abdominalis* (~ 30 PSU). There is some evidence that higher salinity reduces the recovery of dissolved organic matter during the filtration step (SPE C18 cartridges), because salt can change the hydrophobic interaction between the dissolved compounds and the stationary phase (Kruger et al., 2011). Nonetheless, there is also a extraction technique used called the “salting-out effect”, where salt is added to the solution to increase ionic strength and thus extraction yield (Urge et al., 2023), this however is only up to a certain salinity threshold. On a further note, ELISA depend on antibody-antigen binding, with some kits being more sensitive to high ionic conditions (Mukunzi et al., 2017), we therefore also have to consider the possibility that there were some minute salt residues after the first column extraction phase that could have interfered with the immunoassay detection.

**Figure S3.** Gonadosomatic index (GSI) across three syngnathid species. GSI (%) was calculated as ovarian mass divided by total body mass. Sample sizes were *Hippocampus abdominalis* n = 9, *Nerophis ophidion* n = 12, and *Syngnathus typhle* n = 12. *H. abdominalis* exhibited significantly lower GSI compared to both *S. typhle* and *N. ophidion* (Dunn’s test, adj.p < 0.01), whereas no significant difference was observed between *N. ophidion* and *S. typhle*.

**Figure S4: Boxplots of individual hormonal concentrations across species and tissues.**

The following plots are separated by concentration (ng/g tissue) log transformed for each hormone starting with A) Testosterone, B) 11-Keto-Testosterone, C) Estradiol, D) Cortisol, E) Progesterone, F) Prolactin, and G) Isotocin. On the y-axis, the concentration is already normalised to tissue weight and log transformed. Each species separates the x-axis, and each boxplot is colour coded with olive green for liver female, orange for liver male, yellow for ovaries, gray for pouch tissue, and purple for testes.
